# An exploratory study of the effect of age and gender on face scanning during affect recognition in immersive virtual reality

**DOI:** 10.1038/s41598-024-55774-3

**Published:** 2024-03-06

**Authors:** Luz M. González-Gualda, Miguel A. Vicente-Querol, Arturo S. García, José P. Molina, José M. Latorre, Patricia Fernández-Sotos, Antonio Fernández-Caballero

**Affiliations:** 1 Servicio de Salud de Castilla-La Mancha, Complejo Hospitalario Universitario de Albacete, Servicio de Salud Mental, 02004 Albacete, Spain; 2 Neurocognition and Emotion Unit, Instituto de Investigación en Informática de Albacete, 02071 Albacete, Spain; 3 Departmento de Sistemas Informáticos, Universidad de Castilla-La Mancha, 02071 Albacete, Spain; 4 Departmento de Psicología, Universidad de Castilla-La Mancha, 02071 Albacete, Spain; 5 CIBERSAM-ISCIII (Biomedical Research Networking Centre in Mental Health, Instituto de Salud Carlos III), 28016 Madrid, Spain

**Keywords:** Human behaviour, Biomedical engineering

## Abstract

A person with impaired emotion recognition is not able to correctly identify facial expressions represented by other individuals. The aim of the present study is to assess eyes gaze and facial emotion recognition in a healthy population using dynamic avatars in immersive virtual reality (IVR). For the first time, the viewing of each area of interest of the face in IVR is studied by gender and age. This work in healthy people is conducted to assess the future usefulness of IVR in patients with deficits in the recognition of facial expressions. Seventy-four healthy volunteers participated in the study. The materials used were a laptop computer, a game controller, and a head-mounted display. Dynamic virtual faces randomly representing the six basic emotions plus neutral expression were used as stimuli. After the virtual human represented an emotion, a response panel was displayed with the seven possible options. Besides storing the hits and misses, the software program internally divided the faces into different areas of interest (AOIs) and recorded how long participants looked at each AOI. As regards the overall accuracy of the participants’ responses, hits decreased from the youngest to the middle-aged and older adults. Also, all three groups spent the highest percentage of time looking at the eyes, but younger adults had the highest percentage. It is also noteworthy that attention to the face compared to the background decreased with age. Moreover, the hits between women and men were remarkably similar and, in fact, there were no statistically significant differences between them. In general, men paid more attention to the eyes than women, but women paid more attention to the forehead and mouth. In contrast to previous work, our study indicates that there are no differences between men and women in facial emotion recognition. Moreover, in line with previous work, the percentage of face viewing time for younger adults is higher than for older adults. However, contrary to earlier studies, older adults look more at the eyes than at the mouth.Consistent with other studies, the eyes are the AOI with the highest percentage of viewing time. For men the most viewed AOI is the eyes for all emotions in both hits and misses. Women look more at the eyes for all emotions, except for joy, fear, and anger on hits. On misses, they look more into the eyes for almost all emotions except surprise and fear.

## Introduction

Facial expressions are among the major channels of information in interpersonal communication^1^. People infer other people’s basic emotions such as joy, sadness, anger and fear, primarily from facial expressions and tone of voice, with the components of nonverbal communication providing the most information^2^. When there is an impairment in emotion recognition, the person is not able to correctly identify facial expressions represented by other individuals, unavoidably leading to misinterpretation of social cues^3^. All this leads to impairment in interpersonal communication and social functioning^4–6^. This impairment has been described in different neuropsychiatric diseases^7^, schizophrenia being the paradigmatic disease^8^. Patients with schizophrenia present a severe difficulty in facial emotion recognition involving, on the one hand, inaccurate emotional identification and, on the other hand, a negative recognition bias. Inaccuracy involves the inability to accurately recognize facial emotions^9^. Negative bias refers to the identification of neutral faces as negative or threatening stimuli^10^. A recent study analyzing brain responses to scenic stimuli in patients with schizophrenia revealed hyper-reactivity to stimuli evoking high arousal negative emotions and a bias toward fear in the recognition of altered emotions^11^.

Impaired facial recognition of emotion appears to be related, in part at least, to impaired attention to key areas of the face like the eyes, nose, or mouth^12^. The scanning strategy of patients with schizophrenia is characterized by fewer and longer fixations, reduced saccades, and avoidance of relevant facial traits^13,14^. Earlier studies have demonstrated that age influences emotion recognition, with older adults experiencing more difficulty recognizing emotions. Older adults are worse at recognizing sad, angry, and fearful faces as they look longer at the lower half of the face than the upper half of the face^15,16^. The researchers argued that the information might be incomplete for recognizing these emotions because they require a better examination of the eyes. In another paper^17^, older adults were worst at fear, surprise and sadness. While older adults are worse at emotion recognition than younger adults, younger adults do not look longer at the upper half of the face and older adults do not look more time at the lower half^18^. In terms of gender, earlier studies have reported an advantage of women in emotion recognition^19^, and that they pay more attention to the eyes than men, who pay more attention to the mouth than women do (morph of static pictures^20^, static pictures^15^). Another study confirmed the dominance of women over men in labeling emotions, but a non-significant difference was found in tagging negative emotions^21^. Women’s superiority in emotion recognition seems to be maintained across aging^17^.

At this point, it is important to note that alterations in social cognition in patients with schizophrenia are closely related to dysfunction in specific brain areas that play a central role in the processing of social and emotional information. These areas include the prefrontal cortex, the amygdala, and the mirror system, and it is essential to study them when developing intervention strategies aimed at improving social cognition in patients^22,23^. To understand the above, it is worth noting that the prefrontal cortex, located in the frontal region of the brain, plays a fundamental role in regulating higher cognitive functions, including the processing of social information. Individuals with schizophrenia have demonstrated dysfunction in the prefrontal cortex, which may affect their ability to properly interpret and understand social and emotional cues. On the other hand, the amygdala plays a critical role in processing and regulating emotions. Research has confirmed that people with schizophrenia may have changes in the amygdala that interfere with their ability to recognize and respond appropriately to emotional facial expressions^24^. The mirror system, in turn, is a key component of social cognition and is related to the ability to imitate and understand actions and emotions observed in socializing agents. It has been observed that individuals with schizophrenia may experience difficulties in the functioning of the mirror system, which affects their ability to identify a stimulus, recognize it, and generate an appropriate response in the context of effective communication^25^.

These alterations impair the perception, processing, and integration of social and emotional cues in people with schizophrenia to varying degrees. These dysfunctions can manifest as difficulties in interpreting facial expressions, understanding others’ intentions, inferring mental states, and responding appropriately in social contexts. It is important to emphasize that deficits in social cognition not only affect the ability of individuals with schizophrenia to interact effectively in social situations, but also affect their quality of life, social functioning, and overall development. Therefore, it is essential to develop therapeutic approaches that specifically target these deficits and promote the improvement of emotional recognition and social cognition skills in individuals with schizophrenia.

There are different tools to assess facial emotion recognition. Most tools use conventional emotion recognition tasks through photographs or videos, which has been criticized by different authors who argue that photographs do not represent the nature of the facial stimulus^13^ and videos present a lack of validation and have limitations in terms of the duration and format of the scene^26^. In both cases, the stimuli cannot be easily manipulated to match the difficulty of the task^27^. Conventional tasks are limited in capturing the complexity of real-life emotion recognition, as emotions usually take place in complex environments with distractions and often during virtual interactions. At this point one should talk about the concept of presence, understood as the subjective session of “being-there”^28^. A high degree of presence provides the user with the sensation of physical presence and the illusion of interacting and reacting as being in the real world^29^. Dynamic on-screen stimuli (including non-immersive virtual reality, and videos or movies) elicit only a low level of presence in subjects. Therefore, the stimuli do not evoke the feeling of “being there” in subjects, which is necessary to evaluate emotional states in simulated real-world experiences. Moreover, the stimuli are not interactive, so they do not permit subjects to intervene in the scene, which would open the possibility of recognizing emotional states during interactive tasks.

Advances in immersive virtual reality (IVR) have helped to overcome some of these limitations^30^. IVR facilitates the creation of a simulated interactive environment analogous to the real one, with its different scenarios, objects, and beings in real time^31–34^. This generates the sensation of being physically present in it and the possibility of interacting with it. In three-dimensional (3D) environments in which an individual is likely to be dynamically interacting, relevant stimuli may be manipulated and presented in a context that is meaningful to the subject^35^. Research using implicit measures has shown that 3D VR has the potential to elicit emotions, opening new opportunities to the scientific community^36^. In addition, eye-tracking methodologies make it possible to observe in real time how an individual explores the scene presented^26,37^. Although this paper does not compare IVR with classical photo and video stimuli in facial emotion recognition, it is a truism that the use of dynamic avatars for emotion recognition tasks has become widespread in the last 20 years^38–41^. In addition, scientific evidence has highlighted the superiority of IVR over classic photo and video stimuli in facial emotion recognition^42^. This study, carried out by the research team, compared two stimuli: a classical stimulus with photographs, the Penn Emotion Recognition Test (ER-40), with the set of dynamic avatars designed by the team, and showed that the overall accuracy in identifying emotions was greater for the DVF (88.25%) compared to the faces of the ER-40 stimuli (82.60%). The overall accuracy of emotion identification with our DVFs was consistent with similar studies using virtual faces^43–45^. In addition, recent studies indicate that avatars produce better emotional recognition success and increased activity in brain regions involved in emotional processing, compared to natural stimuli^46^.

In the field of psychotherapeutic interventions, two major dimensions that have been the focus of high-impact research in the area of facial emotion recognition and social cognition are computerized interventions and classical stimuli. Each of these modalities has distinct characteristics and approaches therapeutic goals differently. Classical stimulus interventions focus on direct interaction between therapist and patient, using photographs or static images of human faces for facial emotion recognition. In this research, we will implement a computerized intervention specifically based on IVR.

Recently, the potential of IVR as an innovative tool to enhance facial emotion recognition in individuals with schizophrenia has been explored. IVR provides a highly customizable virtual environment that simulates real-life situations and allows individuals to interact with different emotional avatars and scenarios, thereby stimulating their facial emotion recognition abilities^47^. Scientific evidence supports the use of IVR as an effective therapy for addressing social cognition deficits associated with schizophrenia^48^. IVR provides a virtual environment that replicates realistic social situations and uses dynamic avatars to represent different emotional states, allowing patients to practice and improve their emotional and social recognition skills in a safe and controlled environment. In addition, IVR enables real-time assessment of emotions, thoughts, behaviors and physiological responses, allowing therapists to tailor and personalize virtual scenarios to meet the specific needs of individual patients^49,50^. This promotes active and constructive learning where patients take an active role in developing their cognitive and social skills through IVR therapy. Real-time synchronization of movement with other virtual avatars in IVR can have positive effects on affiliative attitudes and behaviors. Research has shown that synchrony in joint action in IVR promotes a greater sense of social closeness with virtual avatars, which contributes to the development of the patient’s social identity and the acquisition of interpersonal skills^24,51^.

In conclusion, scientific evidence confirms that IVR overcomes the limitations of traditional stimuli, such as photos and videos, by providing an immersive and realistic environment that more closely resembles real social situations. This facilitates the transfer of skills acquired in IVR to real-world situations^52^. The customization and individualization of interventions in IVR are essential to address social cognition deficits in individuals with schizophrenia, as virtual scenarios can be adapted to the specific needs of each patient, including difficulty level and emotional intensity, justifying the conduct of this study.

The present exploratory study evaluates eye gaze and facial emotion recognition in a healthy population using dynamic avatars in immersive virtual reality. For the first time, the viewing of each area of interest of the face in IVR is studied by gender and age in seventy-four healthy volunteers after responding to one of the basic emotions represented by dynamic virtual faces. The aim is to identify possible differences in emotion recognition and face scanning across age and gender. Moreover, knowing the eye scanning pattern of healthy people and its correlation with the rate of hits and misses for each emotion is fundamental when it comes to investigate a new immersive emotion recognition task focused on the evaluation and treatment of people with different neuropsychiatric diseases.

## Results

Throughout the article, the following abbreviations will be used where necessary for face areas: forehead (FH), eyes (EY), nose (NS), cheeks (CH), mouth (MT), and background (BG). In addition, the six basic emotions studied and their abbreviations are: anger (ANG), disgust (DIS), fear (FEA), joy (JOY), sadness (SAD), and surprise (SUR), plus the neutral expression (NEU).

### On the effect of age

**Figure 1:**
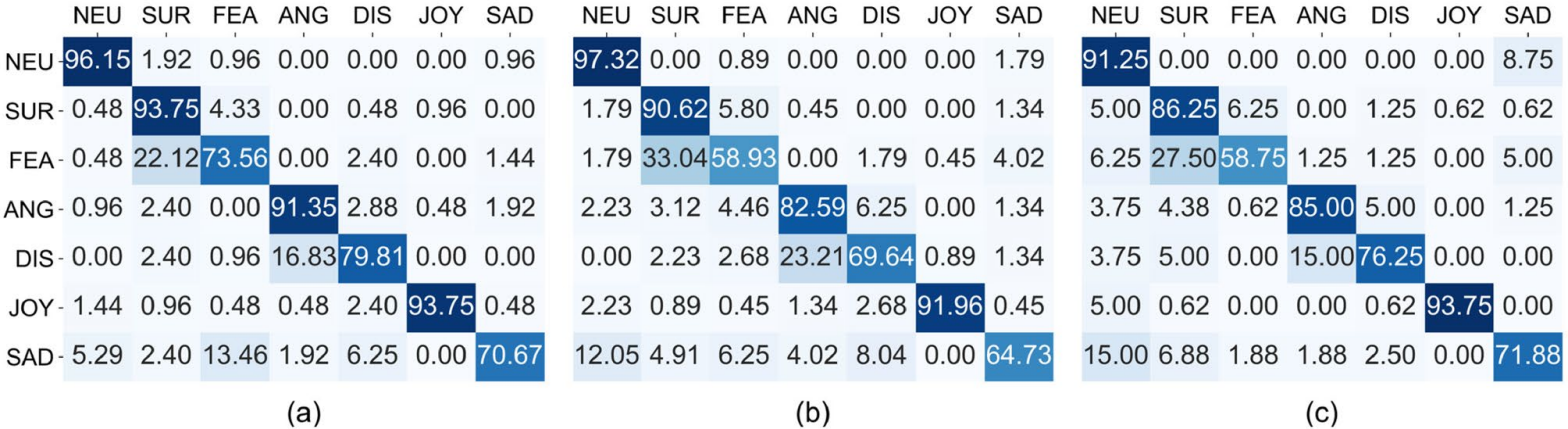
Confusion matrices by age range. (**a**) 20–39, (**b**) 40–59, (**c**) $\ge$ 60 years.

The main diagonal of the age-range confusion matrix (see Fig. 1) shows the percentage of successful identification (hits) of an emotion, while the rows show the percentage when incorrect responses (misses) are selected. The colors of the cells indicate the magnitude of the displayed value. The pattern followed for all age groups is remarkably similar. Despite the variation in the percentages, no statistically significant differences were found in the total number of hits (Kruskal–Wallis $\chi ^2(2)=5.684, p=0.058$). However, talking about individual emotions, this difference is only significant for fear ($\chi ^2(2)=6.465, p=0.039$) and not for neutral ($\chi ^2(2)=0.329, p=0.848$), surprise ($\chi ^2(2)=5.624, p=0.060$), anger ($\chi ^2(2)=4.528, p=0.104$), disgust ($\chi ^2(2)=3.862, p=0.145$), joy ($\chi ^2(2)=1.014, p=0.602$) or sadness ($\chi ^2(2)=5.684, p=0.058$). The Bonferroni post-hoc test was not able to find the differences between age-ranges, although the hit rate is higher for the young group ($73.56\%$ vs. $58.93\%$ and $58.75\%$.

#### Viewing time at each AOI by age

**Figure 2:**
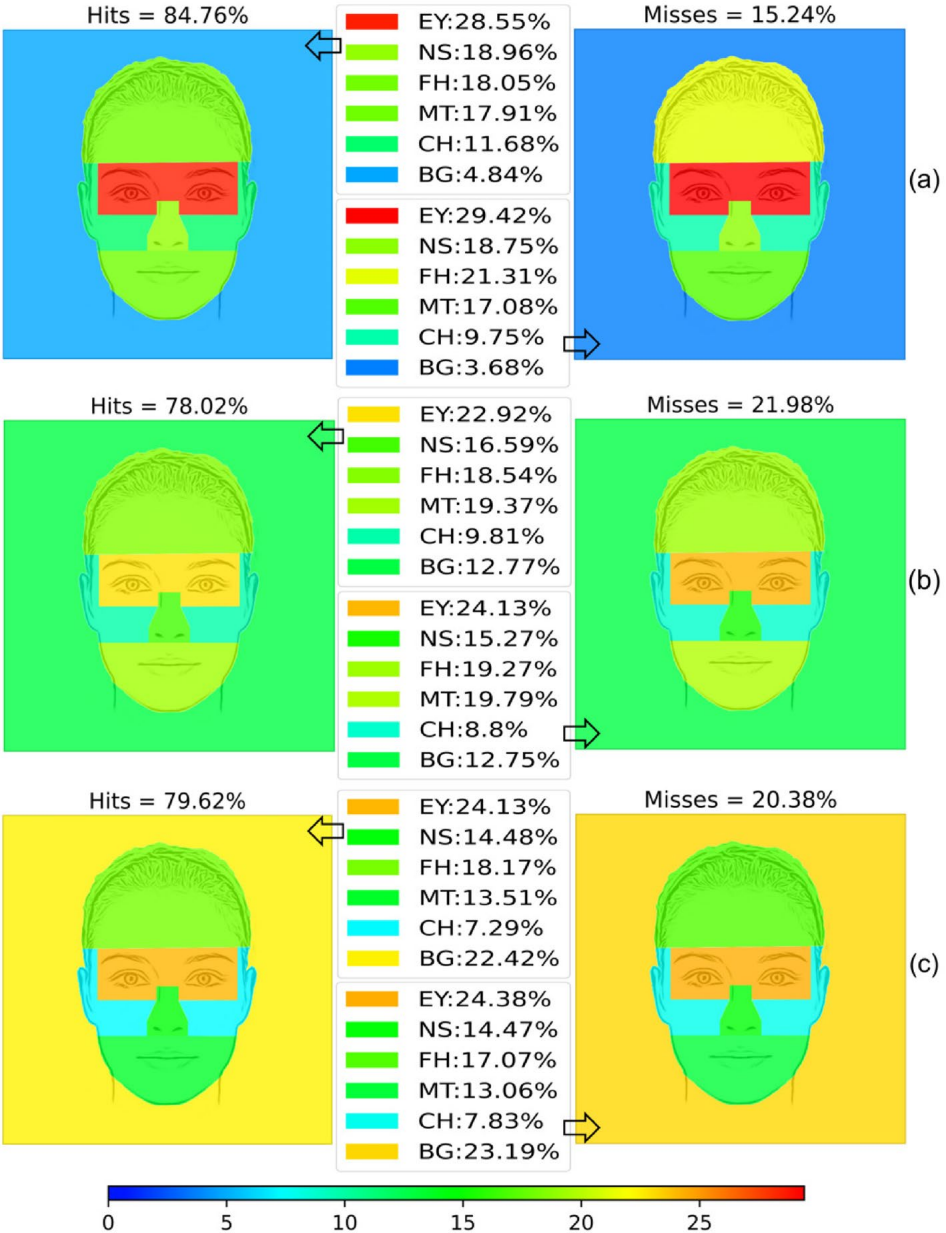
Hits and Misses by age range. (**a**) 20–39, (**b**) 40–59, (**c**) $\ge$ 60 years.

Figure 2 shows the distribution of the percentage of viewing time to decode emotions among the AOIs considered using a heat map representation. Warm colors represent higher viewing times and cool colors represent lower viewing times. , as mentioned in the previous section, there are no differences between groups.

Table 1 summarizes the results of the statistics calculated for the overall number of hits and misses without differentiating between emotions. For each age range, the most viewed AOIs are highlighted first. In addition, statistically significant differences in the time participants spent looking at each part of the face are presented to compare whether they align with the differences found in the percentages (Friedman’s test). Where significant differences were found, the results of pairwise comparisons (indicating which AOIs are viewed more or less than others) are likewise presented.

**Table 1:** Summary of the viewing time results by age group.

Younger adults	Hits	Misses
Most viewed AOIs	EY ($28.55\%$)	EY ($29.42\%$), FH ($21.31\%$)
	NS ($18.96\%$), FH ($18.05\%$), MT ($17.91\%$)	NS ($18.75\%$), MT ($17.08\%$)
Statistical significance	Yes ($\chi ^2(5)=46.615, p<0.001$) for	Yes ($\chi ^2(5)=46.006, p<0.001$) for
	EY > CH ($p=0.013$), EY > FH ($p=0.006$),	EY > MT ($p=0.036$), EY > FH ($p=0.015$),
	EY > BG ($p<0.001$)	EY > CH ($p=0.002$), EY > BG ($p<0.001$)
		BG < FH ($p=0.045$), BG < MT ($p=0.019$),
		BG < NS ($p<0.001$)

Middle-aged adults	Hits	Misses
Most viewed AOIs	EY ($22.92\%$)	EY ($24.13\%$)
	MT ($19.37\%$), FH ($18.54\%$)	MT ($19.79\%$), FH ($19.27\%$)
Statistical significance	Yes ($\chi ^2(5)=22.612, p<0.001$) for	Yes ($\chi ^2(5)=20.951, p=0.001$) for
	EY > CH ($p=0.020$), EY > BG ($p<0.001$)	EY > CH ($p=0.040$), EY > BG ($p=0.001$)

Older adults	Hits	Misses
Most viewed AOIs	EY ($24.13\%$), BG ($22.42\%$)	EY ($24.38\%$), BG ($23.19\%$)
	FH ($18.17\%$)	FH ($17.07\%$)
Statistical significance	Yes ($\chi ^2(5)=14.343, p=0.014$) for	Yes ($\chi ^2(5)=14.483, p=0.013$) for
	EY > CH ($p=0.003$)	EY > CH ($p=0.006$)

Focusing on the differences in performance when there is a hit or a miss, there are no differences in time spent looking at any part of the face, as can be seen in Table 2. Studying the differences in time spent looking at the AOIs for each age group separately, it is noteworthy that the cheeks do not seem to attract so much attention for either group, but their percentage decreases with age (from 11.68 to $7.29\%$). Time spent on the cheeks is the only one that is significantly different between age ranges (Kruskal–Wallis $\chi ^2(2)=8.949, p=0.011$). There is a statistically significant difference between the elderly and the young ($p=0.009$), with the elderly spending significantly less time on hits. It also appears apparent that the three groups spend the highest percentage of time looking at the eyes, but the youngest adults have the highest percentage over the three. However, there are no significant differences in eye gazing time by age group (Kruskal–Wallis $\chi ^2(2)=2.517, p=0.284$).

**Table 2:** Results of the Wilcoxon Signed Rank Test comparing viewing time at each AOI when there is a hit and a miss for each age range.

	EY	MT	NS	FH	CH	BG
Young	$-0.800 (0.909)$	$-0.267 (0.790)$	$-0.114 (0.909)$	$-1.543 (0.123)$	$-1.791 (0.073)$	$-1.714 (0.086)$
Middle-aged	$$-0.745 (0.456)$$	$$-0.336 (0.737) $$	$$-1.586 (0.113)$$	$$-1.333 (0.182)$$	$$-1.033 (0.302)$$	$$-0.368 (0.713)$$
Older	0.224 (0.823)	$$ -0.896 (0.370)$$	$$ -0.523 (0.601)$$	$$-1.006 (0.314)$$	$$-0.149 (0.881)$$	$$-0.373 (0.709) $$

Each cell shows the *Z* value and the *p* value in brackets.

It is also noteworthy that the attention paid to the face relative to the background decreases with age. Younger adults pay more attention to the face when there is a hit ($4.84\%$ of the time it goes to the background), while for middle-aged adults attention to the background increases ($12.77\%$) and for older adults more than $20\%$ of the time they do not look at the face ($22.42\%$). This difference can easily be seen in Fig. 2 by attending to the changes in background color, which ranges from cool (blue) to warm (yellow). However, although there is a difference in the percentages, this difference is not statistically significant (Kruskal–Wallis $\chi ^2(2)=0.742, p=0.690$). When an emotion is not recognized, attention to the background is even higher for middle-aged and older adults ($12.75\%$ and $23.19\%$, respectively). However, and like the case when emotions are correctly identified, this difference is not statistically significant ($\chi ^2(2)=4.4982, p=0.106$).

#### Viewing time at each AOI by emotion and age

Tables A.1, A.2 and A.3 (see Supplementary Material A) show a summary of statistical analysis of the AOI viewing time by emotion and age. In general, the eyes have the largest percentage of viewing time, even when the observer does not recognize the emotion. As stated earlier, young adults look longer at the eyes than the rest of the groups, but it has now become apparent that this is the case for almost all emotions.

Looking at emotions, significant differences were found for fear (Kruskal–Wallis $\chi ^2(2)=6.465, p=0.039$), but the pairwise comparison could not locate them. However, they are close to significant for older versus younger adults ($p=0.088$) and middle-aged versus younger adults ($p=0.086$). Surprise, anger, and neutral hits are more similar, but with a slight decrease with age. For happiness, sadness, anger, and disgust, the decrease in hit rate occurs from young adults ($93.75\%$, $70.67\%$, $91.35\%$ and $79.81\%$) to middle-aged adults ($91.96\%$, $64.73\%$, $82.59\%$ and $69.64\%$), but not from middle-aged to older adults ($93.75\%$, $71.88\%$, $85\%$ and $76.35\%$. For sadness, the hit rate is even slightly higher ($70.67\%$ vs. $71.88\%$), but neither is statistically significant.

### On the effect of gender

**Figure 3:**
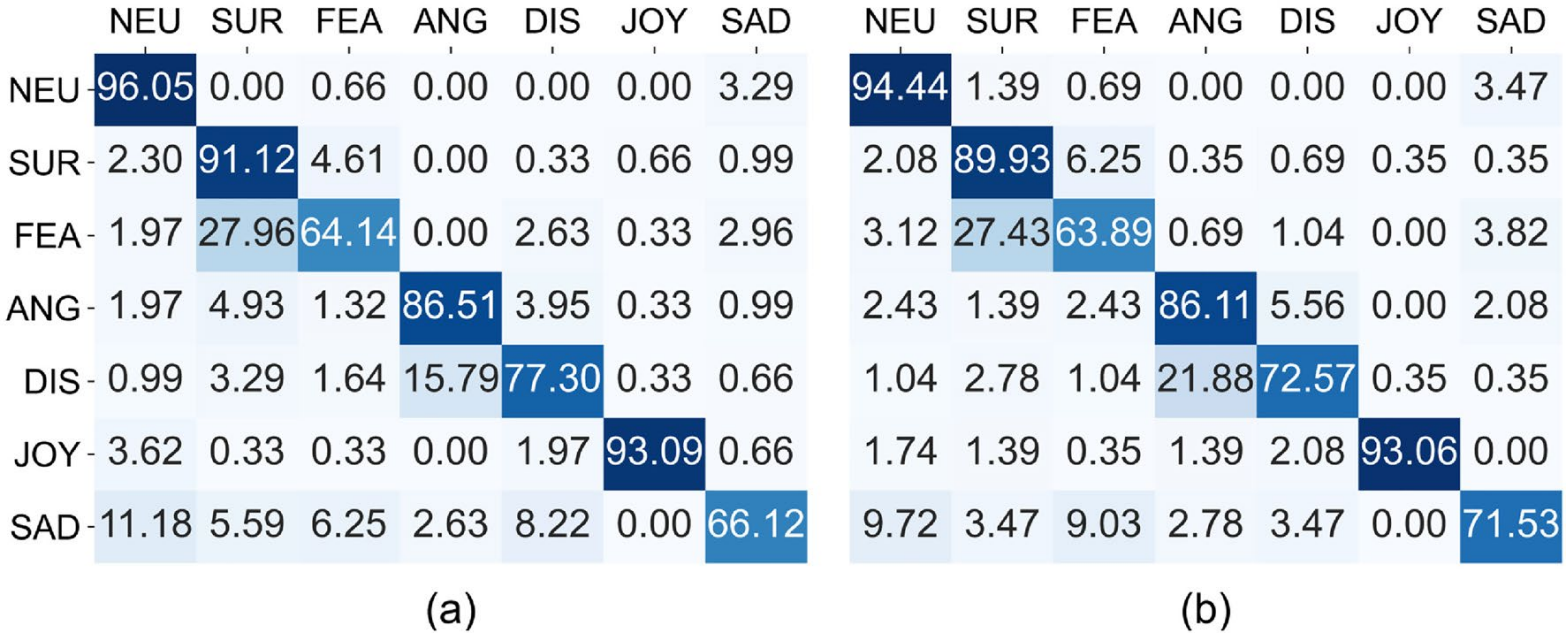
Confusion matrices by gender. (**a**) Men. (**b**) Women.

The confusion matrices for both genders are shown in Fig. 3. The hits between females and males are remarkably similar and, in fact, there are no statistically significant differences between them (Mann-Whitney U Test $U=691.5, p=0.935$). Apart from neutral expression, joy has the highest hit rates (around $93\%$) for both genders, whereas fear has the lowest hit rates (around $64\%$). Surprise and anger have almost the same hits for both genders, while for sadness and disgust the difference between hit rates is higher.

The most noticeable differences between women and men in terms of individual emotions are found in that men decode quite better disgust ($77.30\%$ vs. $72.57\%$) but women decode better sadness ($71.53\%$ vs. $66.2\%$). The most obvious mistakes are that surprise is answered instead of fear (more than $27\%$ both genders) and fear instead of disgust ($15.79\%$ for men and $21.88\%$ for women). Also, importantly, men confuse mainly anger with surprise ($4.93\%$) and disgust ($3.95\%$) and they also confuse sadness mainly with neutral ($11.18\%$) and disgust ($8.22\%$) and a bit less with fear ($6.25\%$), whereas women confuse anger mainly only with disgust ($5.56\%$) and sadness mainly with neutral ($9.72\%$) and fear ($9.03\%$). However, despite the obvious difference on the percentages, there are no statistically significant differences in the number of hits for each emotion and gender, as it is confirmed by the Mann-Whitney U Test for gender and emotion: $U=694.500, p=0.841$ for neutral, $U=636.000, p=0.841$ for surprise, $U=685.500, p=0.987$ for fear, $U=705.000, p=0.811$ for anger, $U=634.000, p=0.578$ for disgust, $U=644.500, p=0.596$ for joy, $U=716.500, p=0.721$ for sadness.

#### Viewing time at each AOI by gender

**Figure 4:**
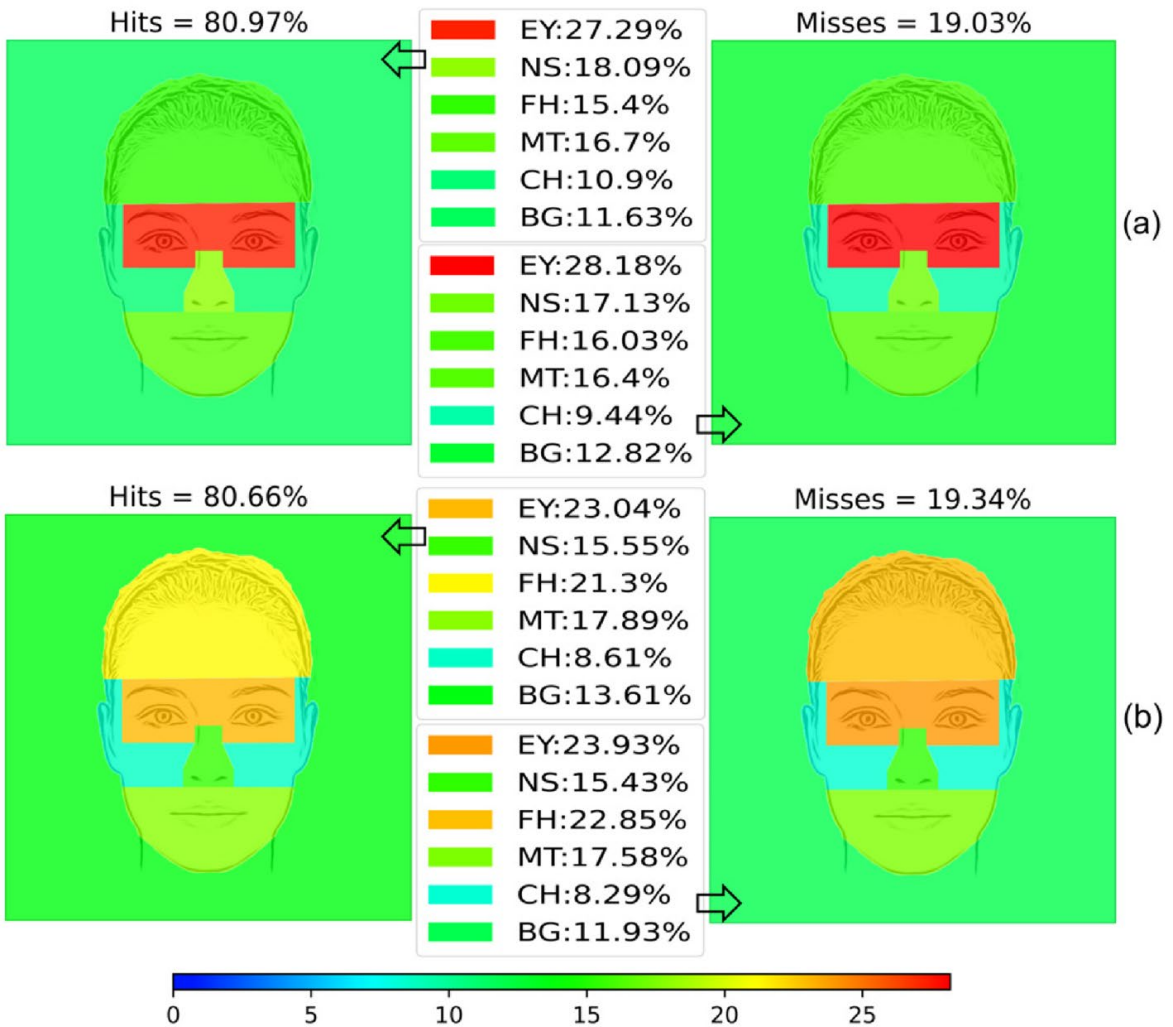
Hits and misses by gender. (**a**) Men. (**b**) Women.

Figure 4 summarizes the average viewing time at each AOI when the emotions were correctly decoded (hit) and when they were not (miss) in a graphical way. Table 3 shows the statistical analysis performed for the overall number of hits and misses.

**Table 3:** Summary of the viewing time results by gender.

Men	Hits	Misses
Most viewed AOIs	EY ($27.29\%$)	EY ($28.18\%$)
	NS ($18.09\%$)	NS ($17.13\%$), MT ($16.40\%$), FH ($16.03\%$)
Statistical significance	Yes ($\chi ^2(5)=43.534, p<0.001$) for	Yes ($\chi ^2(5)=37.481, p<0.001$) for
	EY > FH ($p=0.011$), EY > CH ($p=0.001$),	EY > FH ($p=0.017$), EY > CH ($p=0.002$)
	EY > MT ($p=0.049$), EY > BG ($p<0.001$)	EY > BG ($p<0.001$)
	BG < NS ($p<0.001$), BG < MT ($p=0.014$)	BG < NS ($p=0.008$), BG < MT ($p=0.011$)

Women	Hits	Misses
Most viewed AOIs	EY ($23.04\%$), FH ($21.30\%$)	EY ($23.93\%$), FH ($22.85\%$)
	MT ($17.89\%$), NS ($15.55\%$)	MT ($17.58\%$), NS ($15.43\%$)
Statistical significance	Yes ($\chi ^2(5)=28.762, p<0.001$) for	Yes ($\chi ^2(5)=37.309, p=0.001$) for
	EY > FH ($p=0.037$), EY > CH ($p<0.001$),	EY > FH ($p=0.027$), EY > CH ($p=0.001$),
	EY > BG ($p<0.001$)	EY > MT ($p=0.022$), EY > BG ($p=0.001$)
		NS > BG ($p=0.010$)

Overall, men look longer at the eyes than women ($27.29\%$ vs. $23.04\%$ for hits and $28.18\%$ vs. $23.93\%$ for misses), but women look longer at the forehead and mouth than men. However, there are no statistically significant time differences between genders when there are hits or misses in each AOI, as shown in Table 4.

**Table 4:** Results of the Mann’s Whitney U Test comparing the difference in performance by gender for hits and misses.

	EY	MT	NS	FH	CH	BG
Hits	560.00 (0.180)	679.00 (0.957)	552.00 (0.153)	653.50 (0.741)	525.00 (0.086)	659.00 (0.787)
Misses	657.00 (0.770)	671.00 (0.888)	683.00 (0.991)	604.00 (0.386)	600.00 (0.364)	679.00 (0.948)

Each cell shows the *U* value for the test and the *p* value in brackets.

There are no significant differences in the percentages of women and men looking at each AOI when comparing the results obtained for the hits and misses (see Table 5).

**Table 5:** Results of the Wilcoxon Signed Rank Test comparing viewing time at each AOI when there is a hit and a miss for gender.

	EY	MT	NS	FH	CH	BG
Women	$-1.697 (0.090)$	$-0.456 (0.649)$	$-0.094 (0.925)$	$-0.759 (0.448)$	$-0.660 (0.509)$	$-1.753 (0.080)$
Men	$$-0.445 (0.656)$$	$$-0.475 (0.635)$$	$$-1.411 (0.158)$$	$$-0.990 (0.322)$$	$$-1.697 (0.090)$$	$$-0.445 (0.656) $$

Each cell shows the *Z* value for the test and the *p*-value in brackets.

#### Viewing time at each AOI by emotion and gender

Tables B.1 and B.2 (see Supplementary Material B) show a summary of statistical analysis of the AOI viewing time by emotion and gender. When looking at the tables, it is apparent that men look longer at the eyes than women when there is a hit, and women look longer at the mouth than men in all emotions. However, although there is a difference in the percentages, this difference is not statistically significant.

When an emotion was not correctly classified, the percentage of the background viewing time increased for all emotions. For women, this was only true for neutral, surprise, fear, and joy. When men and women failed to classify an emotion, there was insufficient evidence to suggest that the miss rates tended to closely match those of the emotion they were confused with. However, it appears that the other AOIs were also scrutinized, but the differences were not statistically significant.

## Discussion

### General considerations

As expected, the eyes are the AOI with the highest percentage of viewing time in all cases^53^. Such a difference in looking at the eye region is consistent with the findings in real faces^54–56^. This finding makes sense because clues relevant to emotional information are found in this area^57^. Deepening the fixation time on the eyes, for men the most viewed AOI was the eyes for all emotions, both in the case of hitting and missing. The exception is in the case of misidentifying joy in which more attention was paid to the mouth. For hits, this greater attention on the eyes is statistically significant against forehead, cheeks, mouth, and background for all emotions except fear, for which is only statistically significant against forehead, cheeks, and background. For misses, this attention on the eyes is only statistically significant for fear (forehead and cheeks), anger and disgust (cheeks and background), and sadness (background). In the case of women, the results were more heterogeneous. In case of success, they looked more at the eyes in all emotions except joy, fear, and anger. In case of misses, they looked more at the eyes in almost all emotions except surprise and fear. For hits, this greater attention on the eyes is statistically significant against cheeks and background for all emotions except joy, for which is statistically significant against forehead, cheeks, and background. Whereas for misses, this attention on the eyes is only statistically significant for fear (cheeks, mouth, and background), disgust and sadness (cheeks and background).

Examining the percentage of viewing time on the eyes by age group, it is striking that for the younger group, the most viewed AOI was the eyes for all emotions, both in the case of hits and misses. In the middle-aged group, the most visualized AOI was the eyes for all successfully selected emotions, except for joy where the most visualized AOI was the mouth. For misses, the most attention was not focused on the eyes for most emotions (neutral, surprise, anger, joy, and sadness). Something similar occurred for the older age group. For hits, the most visualized AOI was the eyes for all emotions. In general, for all three age groups and for both hits and misses, the eyes are only statistically significant against cheeks and background.

For both age and gender, these statistical differences suggest that the eyes, besides from being the most viewed AOI, are more important than the previously mentioned AOIs in decoding an emotion. Similar results have been found in recent works not dedicated to age and gender differences. This is the case of a recent registered report shedding light on the psychological mechanisms underlying social face evaluation^58^. The authors found that the single most informative source of information for humans’ trustworthiness ratings of faces are the eyes and eyebrows. Also, a study examined cultural differences in face scanning during dyadic social interactions, revealing greater face orienting during periods of listening compared to speaking^59^.

From our point of view, there are no studies that have studied the percentage of visualization of each AOI in IVR by gender or age, so we cannot compare our results with previously published studies. Hence, more studies will be necessary to clarify the aspects indicated.

### On the effect of gender

No statistically significant differences were found in the number of hits by emotion and gender. earlier studies that have not used virtual reality have shown an advantage of women in emotion recognition^15,20^. These articles concluded that women pay more attention to the eyes than men, who pay more attention to the mouth than women. Other studies also point to a female advantage^60–63^. In contrast to these findings, our study reports that there are no differences between men and women in facial emotion recognition, which is consistent with a more recent work using static pictures^17^. Moreover, Table 3 shows that men look more at the eyes than women and women look more at the mouth than men for all emotions (except fear, sadness, and anger). Apart from this, our results are consistent with earlier studies that have used virtual humans^42,53^. Therefore, the discrepancy with previous work could be explained because of the difference in the stimuli used in each case (classical stimuli versus virtual reality).

The most visualized AOI in both men and women was the eyes. However, men fixated more on the eyes, nose, and cheeks (both in case of success and failure), whereas women focused more on the forehead and mouth (also both in case of success and failure). Although the time spent looking at each AOI was different for men and women, no statistically significant differences were found between the two groups (Table 4, Fig. 4). However, our study shows that men look longer at the eyes than women, women look longer at the forehead, men look longer at the nose, and both look at about the same viewing time at the mouth (see Fig. 4).

If analyzing the visualization time on each AOI by emotion and gender, it can be summarized that there seems to be similarities in the most visualized AOIs for certain emotions in both groups. This is true for fear and surprise (in which the most visualized AOIs were the eyes and the forehead, being the difference between this two AOIs statistically significant in favor of the eyes for men but not for women) and joy (in which the most visualized AOIs were the eyes and the mouth, although the nose was equally important as the mouth in the case of men, been only the difference between eyes and mouth statistically significant for men). For the remaining emotions, there was a different visualization on AOIs for men and women. While for the neutral emotions, anger, disgust and sadness, men paid more attention to the eyes and nose, women gave greater importance to the eyes and forehead. But these differences are not statistically significant.

### On the effect of age

As for the overall hit rate, although the results were similar for all age groups, the young age group obtained the best results (85.57% vs. 79.39% for the middle age group and 80.44% for the older adults). For all groups, the best recognized emotions were neutral expression and positive emotions (joy and surprise), followed by anger. The worst recognized emotions were the negative emotions (disgust, sadness, and fear). Fear is the worst recognized emotion for all three age groups, especially for middle-aged and older adults, where the rate of correct responses dropped to around 59%. Surprise, anger, fear and disgust were better recognized by the younger group, while neutral expression was recognized better by the middle-aged group and sadness was best recognized by the older adults.

As for age, earlier studies showed that age influences emotion recognition, with older adults having more difficulty identifying emotions. Older adults are worse at recognizing sad, angry, and fearful faces because they look longer at the lower than the upper half of the face^15,16^. All these authors argued that the information might be incomplete for recognizing them because those emotions require better examination to the eyes. This lack of attention to the eyes could result in worse facial recognition^64^. In a quite recent paper^17^, older adults were worse on fear, surprise, and sadness. Another work^18^ indicated that while older adults are worse at emotion recognition than younger adults, younger adults do not look longer at the upper half of the face and older adults do not look longer at the part of the lower half. All these studies used static images.

Looking at our data, older people seem to recognize emotions worse than younger adults in general (see Fig. 2). According to the previous paper^18^, although the percentage of face viewing time for younger adults is higher than for older adults, the latter look longer at the eyes than at the mouth (see Fig. 2), in contrast to other studies. Regarding emotions individually, middle-aged, and older adults are the worst at recognizing anger, fear, and disgust (as shown in Fig. 2). Middle-aged adults are worse at recognizing sadness than younger and older adults, but older adults are slightly better than younger adults at recognizing sadness, which contrasts with earlier studies stating that they had the worst performance on anger, fear, and sadness. Regarding the percentage of time spent viewing each emotion, the results shown in Table 1 indicate that for all ages the upper half of the face is more important than the lower half for anger, fear, sadness, surprise, and disgust. This contrasts with earlier studies that found that the lower half is more important for older adults in decoding anger, fear, and sadness. In joy, the lower half is also more important for all ages, which is consistent with earlier studies.

Despite calculating the number of fixations and average fixation time rather than the percentage of viewing time, a paper also estimated that older adults made fewer fixations on the face than younger adults, but of longer duration^16^. The authors concluded that the poor accuracy in recognizing some of the emotions could be due to the lack of certain information. Likewise, the results of this study show that the trend is that older adults have a worse rate of emotion recognition (see Fig. 2). As age increases, face display time decreases (the percentage of background display time increases from 5.45% for younger adults, to 12.28% for middle-aged adults and to 19.53% for older adults).

Most investigation on facial emotion recognition and age has been conducted with natural stimuli. With this kind of stimuli, there seems to be consensus in that facial affect recognition at the age of 60 and above worsens compared to adulthood^15,65^. The emotions of anger, sadness, and fear show the most significant age-related drops^65^. This decline has been linked to older adults paying less attention to relevant areas of the face such as the eyes and mouth^16^.

Studies that have evaluated the role of age in facial emotion recognition through virtual humans are scarce, although suggesting worse performance after reaching the age of 40. A recent study by our research team found a significant difference between the 40–59 and 20–39 age groups, with the number of correct responses being higher for the youngest. In addition, a significant difference was found between the age groups 20–39 years and 40–59 years, and 20–39 years and over 60 years, in favor of the younger age group in both cases^42^. Another study showed that emotion recognition rates decrease with virtual faces, but not with natural faces, in participants over 40 years of age^38^. Although it has been suggested that this could be due to greater exposure to new technologies in younger people, perhaps the use of more dynamic, reality-like stimuli, such as virtual humans, might allow for more sensitive detection of possible impairment in emotion recognition.

In summary, the conclusion is that better emotional recognition is seen in younger adults (20–39 years). By comparing the results of this group with the mean percentage of correct responses of the other two groups together (40–59 years and $\ge$ 60 years), the recognition accuracy would be higher in the young group for all emotions. A comparison between the 3 age groups shows that all emotions are better recognized by the young group except for neutral expression (in which the middle-aged group scores better) and sadness (better recognized in the older group). The highest accuracy in the recognition of sadness by adults over 59 years of age has been obtained in earlier studies^38,66^. It has been suggested that this may be related to a greater presence of sadness in this age group, incidental to the increasing number of unavoidable losses^67^. In addition, it has been shown that older people show more subjective and physiological reactions to sadness-inducing stimuli^68^.

Analyzing the viewing time on each AOI by emotion and age, we can summarize that there seem to be similarities in the most viewed AOIs in certain emotions when participants answer correctly. This is true for joy, where the most viewed AOIs in all 3 groups are the eyes and the mouth (although in the $\ge$ 60 age group, the nose would be as relevant as the mouth). For fear, sadness, anger, and neutral expression, the most visualized AOIs are the eyes and forehead. For the remaining emotions (surprise and disgust) there is only one coincidence for the three age groups: the eyes are the most visualized AOI. Similarities around the viewing time on each AOI for the conditions of success and failure in emotion identification have been found both for the different age groups and for the two genders. This is very striking and raises a question as to whether when a healthy individual fails in emotional identification, this has to do with their ability to interpret that emotion and not with their eye scanning, which, according to our results, would be remarkably like that of a successful individual.

Existing literature suggests that age affects the recognition of affective facial expressions. Gender also appears to play a role in emotion recognition. Unfortunately, little is known about the differences in emotion recognition abilities between males and females across the lifespan, although females show greater abilities from infancy. Virtual reality-based techniques, such as those proposed in this study, may be a promising tool for advancing research on these differences.

### Limitations

This work is exploratory in nature, with the aim of assessing the feasibility of using affect recognition in immersive virtual reality. The sample size used, although sufficient for the purposes of the study, does not allow us to draw conclusions about the relationship between age groups and gender. In any case, it cannot be concluded from the most recent research on the perception of basic emotions in faces that there are differences between age groups and gender. Moreover, most of the work that has investigated this question has done so with completely different experimental models, without the use of virtual reality.

## Methods

### Participants

Seventy-four healthy volunteers were enrolled to participate in this study. The only inclusion criterion was that participants were aged between 20 and 79 years. Exclusion criteria comprised a diagnosis of mental illness, a personal history of medical illness, and a first-degree family history of psychosis. Screening was performed via the Structured Clinical Interview for DSM-IV Axis I Disorders (SCID-I)^69^, which was administered by a team of psychiatrists. Participants with a self-reported neurological diagnosis were also excluded. Recruitment took place in different sociocultural centers, health centers and universities. The mean age was 46.74, $SD = 14.37$. The sample was stratified by gender ($48.65\%$ women, $51.35\%$ men) and age, for which three ranges were considered (20–39 younger adults, 40–59 middle-aged adults and $>59$ older adults). The distribution according to age and gender is shown in Table 6.

**Table 6:** Number of participants by age and gender.

Age	Women	Men	Total
20–39	13	13	26
40–59	12	16	28
> 59	11	9	20
Total	36	38	74

Bearing in mind that this is a preliminary study aimed at verifying the performance of the facial scanning system in the context of virtual reality, the sample size of participants was defined on the basis of previous work^70,71^. We did not aim to carry out a study with a representative sample in terms of gender and age, but we considered that the sample size was sufficient to study the differences in the three age groups and in both sexes. We did not calculate the interaction between the two variables. G*Power software was used to calculate the sensitivity of the sample to the type of non-parametric statistical tests used ^72^. In sensitivity analyses, the critical population $\chi ^2$ and effect size is computed as a function of $\alpha$, $1 - \beta$, and *N*. Considering the non-parametric tests used in the study (Mann-Whitney U, Kruskal–Wallis and Friedman’s test), we calculated the sensitivity for goodness-of-fit tests $\chi ^2$ with 2 degrees of freedom for age groups and calculated the required effect size. With $\alpha$ $err prob = 0.05$, power $(1 - \beta err prob$) = 0.80, total sample $size = 74$ and $Df = 2$, we found a non-centrality parameter $\lambda = 9.63$, critical $\chi ^2 = 5.99$, and effect size $w = 0.36$. Additionally, we calculated the sensitivity for goodness-of-fit tests $\chi ^2$ with 1 degree of freedom for gender and calculated the required effect size. With $\alpha$ $err prob = 0.05$, power $(1 - \beta err prob) = 0.80$, total sample *size* = 74 and $Df = 2$, we found a non-centrality parameter $\lambda = 7.84$, critical $\chi ^2 = 3.84$, and effect size $w = 0.32$.

The participants in this experiment had no previous experience with the dynamic virtual faces (DVFs) used in the evaluation. The study was conducted according to the guidelines of the Declaration of Helsinki and was approved by the Clinical Research Ethics Committee of the Albacete University Hospital Complex (protocol code 2020/12/141; approval date 26 April 2022). Written informed consent was obtained from all subjects participating in the study.

### Experimental setup

The materials used in the experiment were a laptop computer, a gamepad controller and a head mounted display (HMD). The laptop had the following specifications: 17.3” display, Intel Core i7-9750H, 16 GB RAM and NVIDIA RTX2070 Super. The gamepad was used to allow the user to choose among options displayed on a 2D panel within the virtual environment, specifically in terms of the emotions displayed by the digital character. They could select alternatives using either the analog sticks or the digital directional pad interchangeably, and confirm their choice by pressing a button. The HMD chosen for this study was FOVE (https://fove-inc.com/), capable of tracking the wearer’s eyes, allowing it to capture the time spent looking at each part of the virtual human’s face during emotion recognition. It is equipped with a $2560 \times 1440$ pixel WQHD OLED display, a 100-degree field of view and the eye tracking operates at 120 Hz with an accuracy of 1.15 degrees.

**Figure 5:**
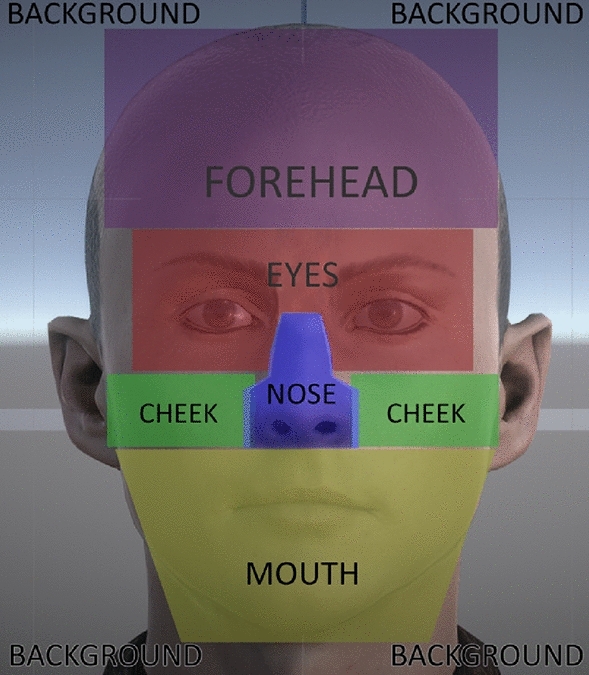
3D geometry and areas of interest in which the viewing time was captured. The eye area includes both eyes and eyebrows.

The face of the virtual humans was divided into different areas of interest (AOIs) as presented in Fig. 5. In addition, the time that participants looked at each AOI was recorded. The figure displays the geometry used to record the time spent looking at each part the virtual humans. When a ray cast from the participants’ eye position intersected with this geometry, the eye gaze time was increased.

### Stimuli

The dynamic virtual faces for facial affect recognition that were used as stimuli in this study represented the six basic emotions anger (ANG), disgust (DIS), fear (FEA), joy (JOY), sadness (SAD), and surprise (SUR), plus the neutral expression (NEU). This set of faces was displayed by 2 Caucasian and 2 African avatars (female and male in both cases) of around 30 years of age, and 2 elderly avatars (female and male). Emotions were designed following the Facial Action Coding System (FACS)^73^. This set of DVFs was recently validated in healthy people^42^ and in people diagnosed with schizophrenia^70^. The description of the process followed to create the virtual humans and emotions was described in detail in^71^. In summary, Adobe Fuse CC was used to create the virtual character models, and Mixamo was used to rig them for body animation. 3D Studio Max was then used to add blendshapes for facial expressions, and the models’ expressions were fine-tuned by hand. Finally, the models were exported to Unity 3D, where wrinkles were added using a custom shader. Although this set was originally designed to be displayed on a computer screen, it has been adapted for immersive virtual reality^74^.

### Procedure

Before starting the study, participants filled out a sociodemographic form (including age, gender, history of mental illness, among others). After filling out the form, participants had to undergo a brief training phase. Calibration of the eye-tracker in which the participant had to look at predefined points for a duration of a couple of minutes was the first task to be completed (this process is automatically executed by the software included with the HMD and is necessary to increase accuracy). Once the calibration was completed, participants were able to familiarize themselves with the controller and the IVR environment under study. Five DVF samples representing random emotions were used for this purpose. A response panel with seven choices (one for each emotion) was displayed after the virtual human represented an emotion.

The experiment supervisor initiated the test when the training phase ended. Each trial began with a random virtual human displaying the neutral emotion and then blended with one of seven random emotions in a process lasting 0.5 s. The emotion was hold for 1.5 s and then blended again into the neutral expression for another 0.5 s. During this process (totaling approximately 2.5 s) the eye-tracker captured which AOIs the participant was looking at and for how long. Once the animation was finished, the response panel appeared, and the participant had unlimited time to answer. Once the response was selected, the entire environment faded to a light gray color and the process resumed. Eye tracking was not active during the process of selecting the appropriate emotion.

Each basic emotion was displayed a total of 8 times with two levels of intensity. The neutral expression only occurred 4 times. Thus, each participant performed altogether 52 trials. In addition, the camera angles varied during the presentation, with a frontal view being used half of the time, and side views (left and right) the other half. The order of face appearances, avatar gender ($50\%$ men and $50\%$ women with slight variations in eye color, skin tone, and hair), race, and age (8 were African and 8 were elderly) were randomized, so the presentation of emotions, camera angles, and virtual humans differed from participant to participant.

### Data analysis

IBM SPSS Statistics v28 was used for statistical analysis. Non-parametric tests were applied due to the nature of the data collected. For hits, only the total amount of hits followed a normal distribution (Kolmogorov–Smirnov 0.093, $p=0.189$), but not the results for hits for the individual emotions (0.500, $p<0.01$ for neutral; 0.301, $p<0.0$1 for surprise; 0.149, $p<0.001$ for fear; 0.215, $p<0.01$ for anger, 0.230; $p<0.01$ for disgust; 0.400, $p<0.01$ for joy and 0.168, $p<0.01$ for sadness). Regarding viewing time, the total viewing time of the eyes, nose and cheeks followed a normal distribution (0.062, $p=0.200$; 0.083, $p=0.200$ and 0.097, $p=0.079$, respectively), while the total viewing time of the front, mouth and background did not (0.193, $p<0.001$; 0.180, $p<0.001$ and = 0.320, $p<0.001$, respectively). Therefore, the Mann-Whitney U test was used to compare hit rates by gender, while Kruskal–Wallis tests were used for comparison of hit rates by age group. The Mann-Whitney U test was used to compare hit rates by gender, while Kruskal–Wallis tests were used for comparison of hit rates by age group. Friedman’s test was employed to compare viewing time between the different AOIs. A *p* value< 0.05 was considered significant. Finally, percentages were used to show hit and miss rates, represented in confusion matrices for the different emotions. Each row of a confusion matrix shows the participants’ responses to the presentation of an emotion. The columns indicate the participants’ response and, therefore, the main diagonal is the percentage of correct responses or hits. The remaining cells in each row show the participants’ wrong responses when an emotion was presented.

### Supplementary Information

Supplementary Information 1.

Supplementary Information 2.

## Data Availability

The datasets generated during and/or analyzed during the current study are available from the corresponding author on reasonable request.
