# Supplementary material for: An exploratory study of the effect of age and gender on face scanning during affect recognition in immersive virtual reality
Source: Sci Rep. 2024 Mar 6;14:5553. doi: 10.1038/s41598-024-55774-3 (PMC10918108; doi:10.1038/s41598-024-55774-3)
Supplement: Supplementary file 1 — Supplementary Information 1. [file 41598_2024_55774_MOESM1_ESM.pdf]

Effect of age and gender on face scanning during affect recognition in immersive virtual reality

Supplementary Material A. Summaries of viewing time results by emotion and age

Table A.1. Summary of the viewing time results by emotion in young adults.

**NEUTRAL**	**Hits**	**Misses**
Most viewed AOIs	EY (30.98%), FH (20.04%)<br>MT (18.12%)	EY (39.09%), MT (27.17%)
Statistical significance	Yes ($\chi^2(5) = 45.885, p < 0.001$) for<br>• EY > FH ($p = 0.002$), EY > CH ($p = 0.002$), EY > MT ($p = 0.013$), EY > BG ($p < 0.001$)<br>• BG < NS ($p = 0.001$), BG < MT ($p = 0.022$)	No ($\chi^2(5) = 7.581, p = 0.181$)
**SURPRISE**	**Hits**	**Misses**
Most viewed AOIs	EY (31.90%)<br>FH (19.09%)	EY (30.67%), FH (24.94%)
Statistical significance	Yes ($\chi^2(5) = 50.501, p < 0.001$) for<br>• EY > FH ($p = 0.006$), EY > CH ($p = 0.003$), EY > MT ($p = 0.028$), EY > BG ($p < 0.001$)<br>• BG < FH ($p = 0.036$), BG < NS ($p < 0.001$), BG < MT ($p = 0.009$)	No ($\chi^2(5) = 7.441, p = 0.190$)
**FEAR**	**Hits**	**Misses**
Most viewed AOIs	EY (28.79%), FH (24.18%	EY (29.78%), FH (20.90%)<br>NS (19.31%)
Statistical significance	Yes ($\chi^2(5) = 41.086, p < 0.001$) for<br>• EY > CH ($p = 0.011$), EY > MT ($p = 0.017$), EY > BG ($p < 0.001$)<br>• BG < FH ($p = 0.007$), BG < NS ($p < 0.001$)	Yes ($\chi^2(5) = 42.298, p < 0.001$) for<br>• EY > MT ($p = 0.031$), EY > BG ($p < 0.001$)<br>• NS > BG ($p < 0.001$)
**ANGER**	**Hits**	**Misses**
Most viewed AOIs	EY (27.50%)<br>FH (19.02%), NS (18.99%)	EY (40.14%)<br>NS (19.37%)
Statistical significance	Yes ($\chi^2(5) = 37.841, p < 0.001$) for<br>• EY > FH ($p = 0.007$), EY > CH ($p = 0.007$), EY > BG ($p < 0.001$)<br>• NS > BG ($p < 0.001$)	No ($\chi^2(5) = 10.630, p = 0.059$)
**DISGUST**	**Hits**	**Misses**
Most viewed AOIs	EY (27.87%)<br>NS (19.48%), MT (18.25%)	EY (28.56%), FH (26.03%), NS (20.22%)
Statistical significance	Yes ($\chi^2(5) = 36.826, p < 0.001$) for<br>• EY > FH ($p = 0.065$), EY > CH ($p = 0.022$), EY > MT ($p = 0.036$), EY > BG ($p = 0.006$)<br>• NS > BG ($p < 0.001$)	Yes ($\chi^2(5) = 23.354, p < 0.001$) for<br>• EY > BG ($p = 0.017$)<br>• NS > BG ($p = 0.031$)
**JOY**	**Hits**	**Misses**
Most viewed AOIs	EY (24.15%), MT (23.13%), NS (21.71%)	EY (30.26%)<br>MT (19.09%), NS (18.86%), CH (18.42%)
Statistical significance	Yes ($\chi^2(5) = 47.560, p < 0.001$) for<br>• EY > FH ($p = 0.001$), EY > BG ($p < 0.001$)<br>• NS > FH ($p = 0.001$), NS > BG ($p < 0.001$)<br>• BG < CH ($p = 0.028$), BG < MT ($p = 0.006$)	No ($\chi^2(5) = 10.541, p = 0.061$)
**SADNESS**	**Hits**	**Misses**
Most viewed AOIs	EY (31.43%)<br>FH (17.70%), NS (17.16%)	EY (24.95%), MT (24.09%)<br>FH (19.72%), NS (18.14%)
Statistical significance	Yes ($\chi^2(5) = 40.909, p < 0.001$) for<br>• EY > FH ($p = 0.028$), EY > CH ($p < 0.001$), EY > MT ($p = 0.022$), EY > BG ($p < 0.001$)<br>• NS > BG ($p = 0.001$)	Yes ($\chi^2(5) = 25.424, p = 0.001$) for<br>• EY > BG ($p = 0.002$)<br>• NS > BG ($p = 0.006$)

Table A.2. Summary of the viewing time results by emotion in middle-aged adults.

**NEUTRAL**	**Hits**	**Misses**
Most viewed AOIs	EY (22.15%), FH (21.23%), MT (20.40%)<br>NS (17.47%)	FH (33.33%), BG (30.44%), MT (23.76%)
Statistical significance	Yes ($\chi^2(5) = 27.304, p < 0.001$) for<br>• EY > BG ($p < 0.001$)<br>• NS > BG ($p = 0.003$)	No ($\chi^2(5) = 2.660, p = 0.752$)
**SURPRISE**	**Hits**	**Misses**
Most viewed AOIs	EY (21.93%)<br>MT (19.76%), FH (19.10%)	FH (29.81%), EY (27.68%)<br>NS (17.31%)
Statistical significance	Yes ($\chi^2(5) = 15.092, p = 0.010$) for<br>• EY > CH ($p = 0.022$), EY > BG ($p = 0.015$)	No ($\chi^2(5) = 8.565, p = 0.128$)
**FEAR**	**Hits**	**Misses**
Most viewed AOIs	EY (24.30%), FH (24.01%)<br>MT (16.65%), BG (14.71%)	EY (26.43%)<br>FH (19.01%)
Statistical significance	Yes ($\chi^2(5) = 24.490, p < 0.001$) for<br>• EY > CH ($p < 0.001$), EY > BG ($p = 0.022$)<br>• FH > CH ($p = 0.036$)	Yes ($\chi^2(5) = 17.658, p = 0.003$) for<br>• EY > BG ($p = 0.003$)
**ANGER**	**Hits**	**Misses**
Most viewed AOIs	EY (24.10%)<br>FH (20.58%), MT (18.58%)	FH (22.92%), EY (21.56%), MT (20.80%)<br>NS (14.03%)
Statistical significance	Yes ($\chi^2(5) = 22.685, p < 0.001$) for<br>• EY > CH ($p = 0.025$), EY > BG ($p < 0.001$)	Yes ($\chi^2(5) = 14.465, p = 0.013$) for<br>• The pairwise comparison did not find differences among the AOIs.
**DISGUST**	**Hits**	**Misses**
Most viewed AOIs	EY (24.27%)<br>NS (17.17%), MT (16.79%), FH (16.65%)	EY (26.40%)<br>FH (19.35%)
Statistical significance	Yes ($\chi^2(5) = 14.774, p = 0.011$) for<br>• EY > BG ($p = 0.006$)	Yes ($\chi^2(5) = 20.313, p < 0.001$) for<br>• EY > CH ($p = 0.009$), EY > BG ($p = 0.005$)
**JOY**	**Hits**	**Misses**
Most viewed AOIs	MT (24.29%)<br>EY (19.89%), NS (18.36%)	BG (41.14%), MT (28.01%)
Statistical significance	Yes ($\chi^2(5) = 21.753, p < 0.001$) for<br>• EY > BG ($p = 0.009$)<br>• BG < NS ($p = 0.012$), BG < MT ($p = 0.032$)	Yes ($\chi^2(5) = 21.086, p < 0.001$)<br>• The pairwise comparison did not find differences among the AOIs.
**SADNESS**	**Hits**	**Misses**
Most viewed AOIs	EY (24.37%)<br>FH (19.82%)	MT (26.43%), EY (20.95%)<br>FH (19.51%)
Statistical significance	Yes ($\chi^2(5) = 25.466, p < 0.001$) for<br>• EY > CH ($p = 0.017$), EY > BG ($p = 0.002$)<br>• NS > CH ($p = 0.046$), NS > BG ($p = 0.005$)	No ($\chi^2(5) = 8.243, p = 0.143$)

Table A.3. Summary of the viewing time results by emotion in older adults.

**NEUTRAL**	**Hits**	**Misses**
Most viewed AOIs	EY (27.55%), FH (20.71%), BG (20.17%)	EY (43.83%), MT (21.75%)
Statistical significance	Yes ($\chi^2(5) = 16.943, p = 0.005$) for<br>• EY > CH ($p = 0.009$), EY > BG ($p = 0.035$)	No ($\chi^2(5) = 5.036, p = 0.412$)
**SURPRISE**	**Hits**	**Misses**
Most viewed AOIs	EY (25.27%), BG (22.04%)<br>FH (19.61%)	BG (30.82%), EY (26.59%)
Statistical significance	Yes ($\chi^2(5) = 14.102, p = 0.015$) for<br>• EY > CH ($p = 0.005$)	No ($\chi^2(5) = 5.911, p = 0.315$)
**FEAR**	**Hits**	**Misses**
Most viewed AOIs	EY (25.44%), FH (21.13%)<br>BG (19.21%)	BG (27.29%), EY (22.42%)
Statistical significance	Yes ($\chi^2(5) = 14.345, p = 0.014$) for<br>• EY > CH ($p = 0.023$)	No ($\chi^2(5) = 10.127, p = 0.072$)
**ANGER**	**Hits**	**Misses**
Most viewed AOIs	EY (24.54%), BG (23.73%)<br>FH (18.64%)	EY (25.90%), BG (24.58%)<br>NS (19.02%)
Statistical significance	No ($\chi^2(5) = 9.832, p = 0.080$)	No ($\chi^2(5) = 9.135, p = 0.104$)
**DISGUST**	**Hits**	**Misses**
Most viewed AOIs	EY (23.04%), BG (22.39%)<br>FH (18.75%), NS (16.35%)	BG (25.80%), FH (25.58%), EY (21.45%)
Statistical significance	Yes ($\chi^2(5) = 16.199, p = 0.006$) for<br>• EY > CH ($p = 0.011$), EY > MT ($p = 0.017$)	No ($\chi^2(5) = 5.342, p = 0.376$)
**JOY**	**Hits**	**Misses**
Most viewed AOIs	EY (24.12%), BG (21.72%)<br>NS (17.20%), MT (17.08%)	BG (27.35%), FH (23.10%)<br>EY (19.69%), MT (19.60%)
Statistical significance	Yes ($\chi^2(5) = 15.635, p = 0.008$) for<br>• EY > CH ($p = 0.007$)	No ($\chi^2(5) = 7.258, p = 0.202$)
**SADNESS**	**Hits**	**Misses**
Most viewed AOIs	BG (22.05%), EY (21.77%)<br>FH (19.64%)	EY (28.56%), BG (25.90%)
Statistical significance	Yes ($\chi^2(5) = 11.422, p = 0.044$) for<br>• The pairwise comparison did not find differences among the AOIs.	Yes ($\chi^2(5) = 12.626, p = 0.027$) for<br>• The pairwise comparison did not find differences among the AOIs.
