# Supplementary material for: An exploratory study of the effect of age and gender on face scanning during affect recognition in immersive virtual reality
Source: Sci Rep. 2024 Mar 6;14:5553. doi: 10.1038/s41598-024-55774-3 (PMC10918108; doi:10.1038/s41598-024-55774-3)
Supplement: Supplementary file 2 — Supplementary Information 2. [file 41598_2024_55774_MOESM2_ESM.pdf]

Effect of age and gender on face scanning during affect recognition in immersive virtual reality

Supplementary Material B. Summaries of viewing time results by emotion and gender

Table B.1. Summary of the viewing time results by emotion in men.

**NEUTRAL**	**Hits**	**Misses**
Most viewed AOIs	EY (27.99%)<br>NS (19.26%)	EY (25.24%), FH (24.14%)<br>BG (19.36%), MT (18.44%)
Statistical significance	Yes ($\chi^2(5) = 45.371, p < 0.001$) for<br>• EY > FH ($p = 0.003$), EY > CH ($p = 0.002$), EY > MT ($p = 0.004$), EY > BG ($p < 0.001$)	No ($\chi^2(5) = 4.512, p = 0.478$)
**SURPRISE**	**Hits**	**Misses**
Most viewed AOIs	EY (29.11%)<br>FH (16.56%), MT (15.96%), NS (15.90%)	EY (27.33%)<br>FH (19.24%), BG (18.42%)
Statistical significance	Yes ($\chi^2(5) = 40.351, p < 0.001$) for<br>• EY > FH ($p = 0.004$), EY > CH ($p < 0.001$), EY > MT ($p = 0.001$), EY > BG ($p < 0.001$)<br>• NS > BG ($p = 0.009$)	No ($\chi^2(5) = 4.382, p = 0.496$)
**FEAR**	**Hits**	**Misses**
Most viewed AOIs	EY (28.94%), FH (20.44%)	EY (28.15%)<br>MT (17.48%), NS (16.88%)
Statistical significance	Yes ($\chi^2(5) = 38.370, p < 0.001$) for<br>• EY > FH ($p = 0.049$), EY > CH ($p < 0.001$), EY > BG ($p < 0.001$)<br>• NS > CH ($p = 0.019$), BG ($p = 0.036$)	Yes ($\chi^2(5) = 33.907, p < 0.001$) for<br>• EY > FH ($p < 0.001$), EY > CH ($p = 0.044$)
**ANGER**	**Hits**	**Misses**
Most viewed AOIs	EY (28.60%)<br>NS (17.43%), MT (16.33%)	EY (34.22%)<br>BG (18.42%)
Statistical significance	Yes ($\chi^2(5) = 40.748, p < 0.001$) for<br>• EY > FH ($p = 0.002$), EY > CH ($p = 0.001$), EY > MT ($p = 0.0264$), EY > BG ($p < 0.001$)<br>• NS > BG ($p = 0.001$)	Yes ($\chi^2(5) = 21.153, p < 0.001$) for<br>• EY > CH ($p = 0.040$), EY > BG ($p = 0.049$)
**DISGUST**	**Hits**	**Misses**
Most viewed AOIs	EY (28.42%)<br>NS (18.42%)	EY (26.35%), FH (20.72%)<br>NS (17.27%)
Statistical significance	Yes ($\chi^2(5) = 40.342, p < 0.001$) for<br>• EY > FH ($p < 0.001$), EY > CH ($p = 0.012$), EY > MT ($p < 0.001$), EY > BG ($p < 0.001$)<br>• NS > BG ($p = 0.002$)	Yes ($\chi^2(5) = 22.363, p < 0.001$) for<br>• EY > CH ($p = 0.006$), EY > BG ($p = 0.024$)
**JOY**	**Hits**	**Misses**
Most viewed AOIs	EY (23.27%), NS (20.79%), MT (20.79%)	BG (33.07%), MT (22.49%)<br>EY (19.36%)
Statistical significance	Yes ($\chi^2(5) = 45.627, p < 0.001$) for<br>• EY > FH ($p = 0.001$), EY > CH ($p = 0.010$), EY > MT ($p = 0.014$), EY > BG ($p < 0.001$)<br>• NS > FH ($p = 0.002$), NS > CH ($p = 0.026$), NS > BG ($p < 0.001$)<br>• MT > BG ($p = 0.006$)	Yes ($\chi^2(5) = 11.269, p = 0.046$) for<br>• The pairwise comparison did not find differences among the AOIs.
**SADNESS**	**Hits**	**Misses**
Most viewed AOIs	EY (27.02%)<br>NS (17.61%), FH (16.64%), MT (16.37%)	EY (27.50%)<br>MT (19.01%)
Statistical significance	Yes ($\chi^2(5) = 42.682, p < 0.001$) for<br>• EY > CH ($p < 0.001$), EY > BG ($p < 0.001$)<br>• BG < NS ($p < 0.001$), BG < FH ($p = 0.049$), BG < MT ($p = 0.021$)	Yes ($\chi^2(5) = 18.648, p = 0.002$) for<br>• EY > BG ($p = 0.014$)

Table B.2. Summary of the viewing time results by emotion in women.

**NEUTRAL**	**Hits**	**Misses**
Most viewed AOIs	EY (25.24%), FH (24.15%)<br>MT (18.44%)	MT (31.72%), FH (25.00%)<br>EY (19.97%)
Statistical significance	Yes ($\chi^2(5) = 38.713, p < 0.001$) for<br>• EY > CH ($p < 0.001$), EY > BG ($p < 0.001$)	No ($\chi^2(5) = 4.815, p = 0.439$)
**SURPRISE**	**Hits**	**Misses**
Most viewed AOIs	EY (23.40%), FH (22.06%)<br>MT (18.62%)	EY (28.61%), FH (27.02%)<br>NS (13.62%), MT (12.88%), BG (12.73%)
Statistical significance	Yes ($\chi^2(5) = 26.758, p < 0.001$) for<br>• EY > CH ($p < 0.001$), EY > BG ($p < 0.001$)	Yes ($\chi^2(5) = 11.789, p = 0.038$) for<br>• The pairwise comparison did not find differences among the AOIs.
**FEAR**	**Hits**	**Misses**
Most viewed AOIs	FH (26.30%), EY (24.74%)<br>MT (16.53%)	EY (28.15%)<br>NS (15.31%), MT (14.23%)
Statistical significance	Yes ($\chi^2(5) = 30.395, p < 0.001$) for<br>• EY > CH ($p = 0.001$), EY > BG ($p < 0.001$)<br>• NS > BG ($p = 0.014$)	Yes ($\chi^2(5) = 31.029, p < 0.001$) for<br>• EY > CH ($p = 0.004$), EY > MT ($p = 0.018$), EY > BG ($p < 0.001$)<br>• NS > BG ($p = 0.025$)
**ANGER**	**Hits**	**Misses**
Most viewed AOIs	FH (24.09%), EY (22.04%)<br>MT (16.37%)	MT (21.95%), EY (20.12%)<br>NS (19.24%)
Statistical significance	Yes ($\chi^2(5) = 23.9718, p < 0.001$) for<br>• EY > CH ($p = 0.002$), EY > BG ($p < 0.001$)	Yes ($\chi^2(5) = 12.503, p = 0.029$) for<br>• The pairwise comparison did not find differences among the AOIs.
**DISGUST**	**Hits**	**Misses**
Most viewed AOIs	EY (21.78%), FH (20.91%)<br>MT (17.93%), NS (17.09%)	EY (25.55%), FH (25.38%)<br>NS (16.06%), MT (15.24%)
Statistical significance	Yes ($\chi^2(5) = 22.266, p < 0.001$) for<br>• EY > CH ($p = 0.002$), EY > BG ($p = 0.006$)	Yes ($\chi^2(5) = 20.726, p < 0.001$) for<br>• EY > CH ($p = 0.037$), EY > BG ($p = 0.011$)
**JOY**	**Hits**	**Misses**
Most viewed AOIs	MT (23.14%), EY (21.75%)<br>NS (17.57%)	MT (23.94%), FH (21.53%), BG (20.40%)
Statistical significance	Yes ($\chi^2(5) = 27.424, p < 0.001$) for<br>• EY > FH ($p = 0.006$), EY > CH ($p = 0.020$), EY > BG ($p < 0.001$)<br>• BG < NS ($p = 0.037$), BG < MT ($p = 0.046$)	No ($\chi^2(5) = 2.573, p = 0.766$)
**SADNESS**	**Hits**	**Misses**
Most viewed AOIs	EY (25.30%), FH (21.39%)	MT (23.64%), FH (21.46%), EY (21.68%)
Statistical significance	Yes ($\chi^2(5) = 27.253, p < 0.001$) for<br>• EY > CH ($p < 0.001$), EY > BG ($p < 0.001$)	Yes ($\chi^2(5) = 19.981, p = 0.001$) for<br>• EY > CH ($p = 0.046$), EY > BG ($p = 0.009$)
